# Domain-Swapped Dimer of *Pseudomonas aeruginosa* Cytochrome *c*_551_: Structural Insights into Domain Swapping of Cytochrome *c* Family Proteins

**DOI:** 10.1371/journal.pone.0123653

**Published:** 2015-04-08

**Authors:** Satoshi Nagao, Mariko Ueda, Hisao Osuka, Hirofumi Komori, Hironari Kamikubo, Mikio Kataoka, Yoshiki Higuchi, Shun Hirota

**Affiliations:** 1 Graduate School of Materials Science, Nara Institute of Science and Technology, 8916–5 Takayama, Ikoma, Nara 630–0192, Japan; 2 Department of Life Science, Graduate School of Life Science, University of Hyogo, 3-2-1 Koto, Kamigori-cho, Ako-gun, Hyogo 678–1297, Japan; 3 Faculty of Education, Kagawa University, 1–1 Saiwai-cho, Takamatsu, Kagawa 760–8522, Japan; 4 RIKEN SPring-8 Center, 1-1-1 Koto, Sayo-cho, Sayo-gun, Hyogo 679–5148, Japan; NCI-Frederick, UNITED STATES

## Abstract

Cytochrome *c* (cyt *c*) family proteins, such as horse cyt *c*, *Pseudomonas aeruginosa* cytochrome *c*_551_ (PA cyt *c*_551_), and *Hydrogenobacter thermophilus* cytochrome *c*_552_ (HT cyt *c*_552_), have been used as model proteins to study the relationship between the protein structure and folding process. We have shown in the past that horse cyt *c* forms oligomers by domain swapping its C-terminal helix, perturbing the Met–heme coordination significantly compared to the monomer. HT cyt *c*_552_ forms dimers by domain swapping the region containing the N-terminal α-helix and heme, where the heme axial His and Met ligands belong to different protomers. Herein, we show that PA cyt *c*_551_ also forms domain-swapped dimers by swapping the region containing the N-terminal α-helix and heme. The secondary structures of the M61A mutant of PA cyt *c*_551_ were perturbed slightly and its oligomer formation ability decreased compared to that of the wild-type protein, showing that the stability of the protein secondary structures is important for domain swapping. The hinge loop of domain swapping for cyt *c* family proteins corresponded to the unstable region specified by hydrogen exchange NMR measurements for the monomer, although the swapping region differed among proteins. These results show that the unstable loop region has a tendency to become a hinge loop in domain-swapped proteins.

## Introduction

In domain swapping, a protein molecule exchanges its secondary or tertiary structural unit with the corresponding unit of another molecule of the same protein [1,2]. Approximately 5% of protein families based on protein structural classification [3] have been found to contain domain-swapped structures [4]. Domain swapping has also been reported in human pathology-related proteins such as serpin, β_2_-microglobulin, and prion [5,6,7]. Diversity of domain swapping exist in the size, sequence, and secondary structure of proteins, and thus it has been proposed that any protein can swap its unconstrained, partial structure(s) under appropriate conditions [4,8,9].

**Fig 1:**
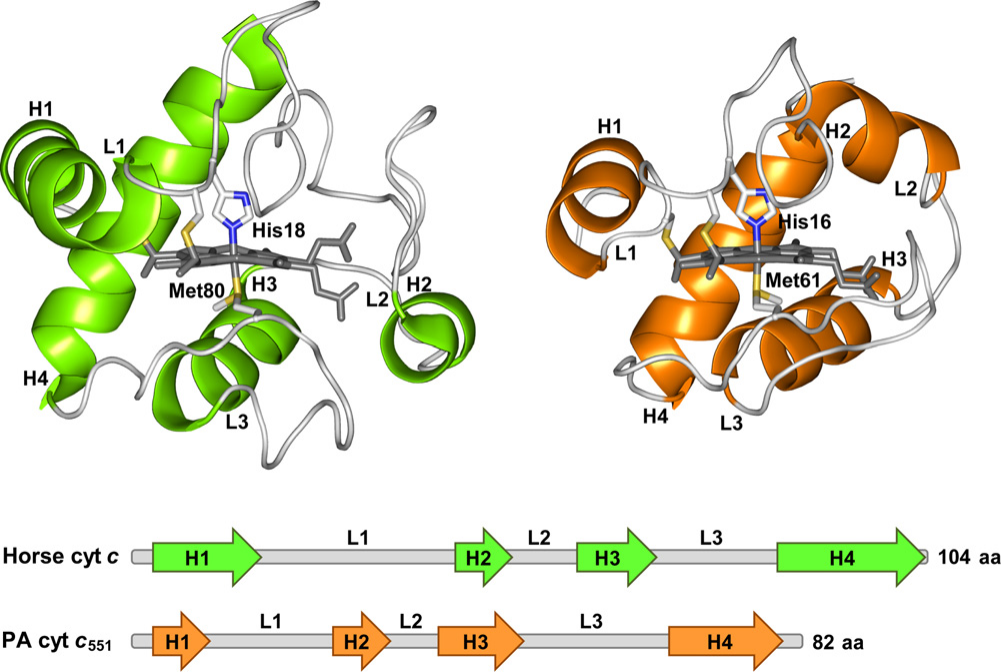
Structures of horse cyt *c* and PA cyt *c*_551_. Horse cyt *c* (upper left) and PA cyt *c*_551_ (upper right). The hemes and axial ligands are shown as stick models. The heme, the sulfur atoms of the heme axial Met ligand and heme-linked Cys, and the nitrogen atoms of the heme axial His ligand are shown in gray, yellow, and blue, respectively. The secondary structure diagrams of horse cyt *c* and PA cyt *c*_551_ are depicted at the bottom of the figure. The helices are depicted as arrows in the secondary structure diagrams. The helices and loops are labeled as H1–H4 and L1–L3, respectively.

Cytochrome *c* (cyt *c*) is an electron transfer protein existing in the inner membrane space of mitochondria. Cyt *c* also plays a key role in apoptosis, where it is released to the cytosol when permeabilization of the mitochondrial outer membrane occurs [10,11]. Cyt *c* contains three long α-helices (helices 1, 3, and 4) and a short α-helix (helix 2). A hexacoordinated heme is attached covalently to two Cys residues through their sulfur atoms in cyt *c* (Fig 1 and Table 1). His and Met residues are coordinated to the heme iron of cyt *c* in its native state [12,13,14]. We have shown that horse cyt *c* forms polymers from monomers by domain swapping its C-terminal α-helix successively [15]. The C-terminal α-helix of dimeric horse cyt *c* was displaced from its original position in the monomer, and the Met–heme coordination was perturbed significantly in the dimer, causing higher cyanide ion binding affinity and peroxidase activity compared to those in the monomer [15,16,17]. *Hydrogenobacter thermophilus* cytochrome *c*_552_ (HT cyt *c*_552_) is a member of the cyt *c* protein family. We have shown that HT cyt *c*_552_ forms oligomers by domain swapping its N-terminal region containing the heme [18]. *Psudomonas aeruginosa* cytochrome *c*_551_ (PA cyt *c*_551_) is also a member of the cyt *c* protein family, and is considered to transfer electrons in the bacterial periplasm. Similar to other cyt *c* proteins, PA cyt *c*_551_ contains four α-helices (helices 1–4), and His16 and Met61 are coordinated to its heme iron (Fig 1 and Table 1) [19].

**Table 1:** Regions of secondary structures of horse cyt *c* and PA cyt *c*_551_.

Secondary structural element	Residues	
	Horse cyt *c* ^a^	PA cyt *c*_551_ ^b^
Helix 1 (N-terminal α-helix)	2–15	3–10
Loop 1	20–49	17–26
Helix 2	(49–54)^c^	27–33
Loop 2	56–60	34–39
Helix 3	61–70	40–49
Loop 3	70–85	50–67
Helix 4 (C-terminal α-helix)	87–104	68–80

^a^ From ref. [20].

^b^ From ref. [21].

^c^ This region has been defined as part of the loop in ref. [20], but represented as helix 2 in solution [14] and X-ray [13] structures.

Cyt *c* family proteins have been used as models for folding studies [20,22,23,24]. Mitochondrial and bacterial cyt *c* proteins have a common folding mechanism, where the N- and C-terminal helices interact with each other in the folding intermediate [22,23,25]. However, there are intriguing differences in the folding and unfolding properties among cyt *c* family proteins. Hydrogen exchange NMR measurements have shown that horse cyt *c* and PA cyt *c*_551_ are composed of five subglobally cooperative unfolding units, called foldons [21,24]. The thermodynamic properties of foldons are represented by the free energy (Δ*G*_HX_) of the structural opening reaction, allowing amide hydrogens protected by hydrogen bonding to exchange with solvent hydrogens. The locations of low energy foldons are different between horse cyt *c* and PA cyt *c*_551_, suggesting different folding processes between them [20,21]. The Met–heme coordination of horse cyt *c* is disrupted at mild denaturing conditions [26] or alkaline pH [27], whereas that of PA cyt *c*_551_ is conserved until the protein is almost completely unfolded [28]. The thermostability of three loop regions (loops 1–3, Fig 1 and Table 1) are different between horse cyt *c* and PA cyt *c*_551_, where the loop containing the heme-ligating Met (loop 3) is more mobile and less stable in horse cyt *c* compared to PA cyt *c*_551_ [20,29]. The differences in the local stability and unfolding property of loop 3 among the cyt *c* family proteins have also been explained by the folding energy landscape [21].

It has been suggested that proteins form domain-swapped oligomers via their partially unfolded structures [30,31,32]. Partial unfolding of a protein is promoted at low pH, high temperature, and in the presence of alcohols [33]. Refolding experiments of ribonuclease A [34] and molecular dynamics simulations of γ-crystallin [35] have revealed that these proteins form domain-swapped oligomers via the folding intermediates possessing regions with native-like structures. We have reported that horse cyt *c* forms domain-swapped oligomers by the interaction between the N- and C-terminal α-helices at the early stage of folding from its unfolded state [36], and the interaction important for formation of domain-swapped oligomers exists in the molten globule state [37]. The hinge loop, a segment of the polypeptide chain that links the swapped domain and the rest of the protein, plays an important role in stabilizing the domain-swapped conformation [4]. The flexibility [8,9] and length [8,9] of the hinge loop, and the structurally weak regions in the protein [38] have been suggested to correlate with the swapping region. However, it is still difficult to predict the swapping region in proteins. In this study, we show that PA cyt *c*_551_ forms oligomers, in which the region containing the N-terminal α-helix and heme are swapped. The swapping region in cyt *c* family proteins is shown to correlate with the less stable local structure, based on the comparison of the structure and folding properties between the monomer and domain-swapped dimer.

## Materials and Methods

### Preparation of cytochrome *c*_551_

Enzymes for site-directed mutagenesis were obtained from Takara Shuzo Co. (Kyoto, Japan). Oligonucleotide primers were purchased from Sigma-Aldrich Japan (Tokyo, Japan). The *E*. *coli* expression system of PA cyt *c*_551_ was gifted from Prof. Sambongi [39]. Amino acid substitution of Met61 was performed by PCR-based *in vitro* mutagenesis of the original plasmid vector using PA-M61A-F and PA-M61A-R primers (S1 Table). Mutated DNA was purified using the QIAprep spin Mini prep kit (QIAGEN, Venlo, Netherlands). DNA sequencing was carried out with the BigDye Terminator v3.1 cycle sequencing kit (Applied Biosystems, Inc., Foster City, CA) and an ABI 3100 Avant generic analyzer (Applied Biosystems, Inc.). Recombinant wild-type (WT) and M61A PA cyt *c*_551_ were overproduced in *E*. *coli* JCB387 cells [40]. The cells were grown at 37°C in 5 L flasks containing 2 L of LB broth (Sigma-Aldrich, St. Louis, USA) (20 g/L) for 12 hours and harvested. Oxidized PA cyt *c*_551_ was purified by the previous method [39]. The purity of oxidized WT and M61A PA cyt *c*_551_ was confirmed by the absorption spectrum and elution curve of gel chromatography. The absorption coefficients of oxidized monomeric WT and M61A PA cyt *c*_551_ were estimated as ε_409_ = 106.1 mM^-1^cm^-1^ and ε_401_ = 142.0 mM^-1^cm^-1^, respectively, by the pyridine hemochrome method [41]. The absorption coefficient of oxidized dimeric WT PA cyt *c*_551_ was estimated as ε_409_ = 109.4 mM^-1^cm^-1^ from the absorbance change by dissociation of the dimer to monomers by heating at 70°C for 10 min. The concentrations of oxidized WT and M61A PA cyt *c*_551_ were calculated from the absorbance at 409 and 401 nm, respectively, and adjusted to desired concentrations.

### Preparation of dimer

PA cyt *c*_551_ precipitates were produced by an addition of 80% (v/v) ethanol to 1 mM oxidized WT or M61A PA cyt *c*_551_. The precipitate was separated from the supernatant by centrifugation, and lyophilized to remove residual ethanol. The obtained precipitate was dissolved with 1 ml of 50 mM potassium phosphate buffer, pH 7.0, at 4°C. Oligomer formation of PA cyt *c*_551_ was analyzed by gel chromatography (Superdex 75, GE healthcare) using a fast protein liquid chromatography (FPLC) system (BioLogic DuoFlow 10, Bio-Rad, CA) at 4°C. WT PA cyt *c*_551_ dimer was purified by repeating gel chromatography (HiLoad 26/60 Superdex 75, GE healthcare) using the FPLC system (BioLogic DuoFlow 10, Bio-Rad) with 50 mM potassium phosphate buffer, pH 7.0. Purified PA cyt *c*_551_ dimer was used immediately after purification.

### Optical absorption and CD measurements

Absorption spectra were measured with a UV-2450 spectrophotometer (Shimadzu, Japan) using a 1-cm path-length quartz cell. CD spectra were measured with a J-725 circular dichroism spectropolarimeter (Jasco, Japan) using a 0.1-cm path-length quartz cell.

### X-ray crystallography

Crystallization of domain-swapped dimeric PA cyt *c*_551_ was carried out at 277 K using the sitting drop vapor diffusion method with Crystal Screen 1 (Emerald Biosystems Inc., Bainbridge Island, USA). The protein concentration was adjusted to 9.2 mg/ml in 50 mM potassium phosphate buffer, pH 7.0. The droplets prepared by mixing 2 μl of the protein solution with 2 μl reservoir solution were equilibrated. The best reservoir solution was found to be 0.1 M HEPES-NaOH buffer, pH 7.5, containing 1.4 M sodium citrate tribasic dehydrate. A crystal was observed in the protein solution after incubation at 4°C for 5 weeks.

The diffraction data were collected at the BL38B1 beamline of SPring-8, Japan. The crystal was mounted on a cryo-loop without an additional cryoprotectant, and flash-frozen at 100 K in a nitrogen cryo-system. The detector was Quantum315 (ADSC). The crystal-to-detector distance was 250 mm and the wavelength was 0.8 Å. The oscillation angle was 0.5° and the exposure time was 3 sec per frame. The total number of frames was 180. The diffraction data were processed using the program HKL-2000 [42].

The preliminary structure was obtained by the molecular replacement method (MOLREP [43]) using the atomic coordinates of the monomer structure of PA cyt *c*_551_ (PDB ID: 351C) as a starting model. There were two protomers, each from a different domain-swapped dimer molecule, in the asymmetric unit of the crystal. The structure refinement was performed using the program REFMAC [44]. The molecular model was manually corrected, and water molecules were picked up in the electron density map using the program COOT [45]. The data collection and refinement statistics are summarized in S2 Table.

### Small angle X-ray scattering measurements

All samples were prepared in 50 mM potassium phosphate buffer, pH 7.0. Small angle X-ray scattering (SAXS) measurements were carried out using a rotating anode X-ray generator, UltraX18 (Rigaku, Tokyo, Japan), in which a monochromatic X-ray with a wavelength of 1.54 Å was focused through a confocal Max-Flux mirror (Rigaku). Scattering profiles were collected using an X-ray image intensifier CCD detector (Hamamatsu Photonics K.K., Shizuoka, Japan). The path length of the sample cell was 1 mm, and its temperature was controlled to 20°C. A series of monomer dilutions were measured to extrapolate the scattering intensity to zero protein concentration and eliminate the inter-particle interference.

### Differential scanning calorimetry measurements

Differential scanning calorimetry (DSC) thermograms of oxidized monomeric and dimeric PA cyt *c*_551_ were measured at a scan rate of 1°C/min with VP-DSC (MicroCal, GE Healthcare) in 50 mM potassium phosphate buffer, pH 7.0.

### Electrochemistry

Cyclic voltammetry responses were obtained with ALS-612DN (BAS Inc., Tokyo, Japan). An Au electrode was used as a working electrode, and Pt wire and Ag/AgCl (3 M NaCl) were used as counter and reference electrodes, respectively. The redox potentials were calculated with respect to the normal hydrogen electrode (NHE). Modification of the surface of the Au electrode was performed by the following procedure [46]. The surface of the Au electrode was polished with 0.05 μm alumina water slurry and subsequently rinsed with pure water. To remove residual organic compounds from the electrode surface, the Au electrode was cleaned by electrochemical oxidation/reduction treatment. The Au electrode was dipped in a methanol solution containing 1 mM 4-mercaptopyridine (Wako, Osaka, Japan) for 30 s, and then rinsed with pure water. Cyclic voltammograms of oxidized monomeric (~100 μM, heme unit) and dimeric (~200 μM, heme unit) PA cyt *c*_551_ were recorded in 50 mM potassium phosphate buffer, pH 7.0, containing 200 mM NaCl (Wako). All the measurements were performed at room temperature after degassing with a vacuum line, and flowing Ar gas for at least 5 min to remove oxygen dissolved in the solution.

## Results

### Oligomer formation of cytochrome *c*_551_

Dimeric, trimeric, and tetrameric WT PA cyt *c*_551_ were produced from oxidized monomeric cyt *c*_551_ by an addition up to 80% (v/v) ethanol, subsequent lyophilization, and resolvation with buffer (S1 Fig). No oligomers larger than the tetramer formed, similar to the case of HT cyt *c*_552_ [18], whereas high order oligomers have been produced in horse cyt *c* by the treatment with ethanol [15]. These results suggest that a structural restriction and/or electrostatic repulsion suppresses formation of high order oligomers for PA cyt *c*_551_. The WT PA cyt *c*_551_ dimer was stable at 4°C, although it converted to monomers when heated at 70°C for 10 min (S1 Fig).

### Structure of dimeric cytochrome *c*_551_

The absorption spectrum of oxidized dimeric WT PA cyt *c*_551_ was similar to that of its monomer (S2 Fig). The 695-nm band characteristic for the Met–heme iron coordination was observed in the spectrum of the dimer, and exhibited a similar intensity as that in the monomer spectrum (S2 Fig). The intensities of the negative 208-nm and 222-nm CD bands in the dimer spectrum were also similar to those in the monomer spectrum (S3 Fig). These results indicate that the heme environment, especially the His and Met coordination to the heme iron and the secondary structures of PA cyt *c*_551_ did not change by the dimerization. These spectral properties were similar to those of HT cyt *c*_552_ [18]. For horse cyt *c*, the intensities of the 695-nm absorption band and the 208-nm and 222-nm CD bands were decreased and increased, respectively, by the dimerization [15], suggesting that the effect of domain swapping on the secondary structures of PA cyt *c*_551_ is similar to that of HT cyt *c*_552_ but different from that in horse cyt *c*.

**Fig 2:**
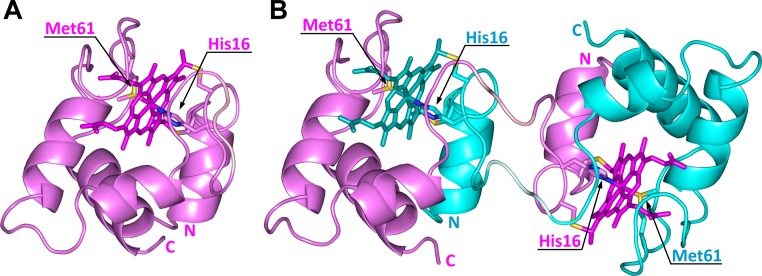
Crystal structures of monomeric and dimeric WT PA cyt *c*_551_. (A) Structure of monomeric WT PA cyt *c*_551_ (PDB ID: 351C). (B) Structure of dimeric WT PA cyt *c*_551_ solved in this study (pink and cyan, PDB ID: 3X39). The two protomers are depicted in pink and cyan, respectively. The hemes, Cys12, Cys15, His16, and Met61 are shown as stick models. The N- and C-termini are labeled as N and C, respectively. The hemes and Thr20–Met22 residues (hinge loop) are depicted in dark and pale colors, respectively. The sulfur atoms of the heme axial Met ligand and heme-linked Cys are shown in yellow, and the nitrogen atoms of the heme axial His ligand are shown in blue.

**Fig 3:**
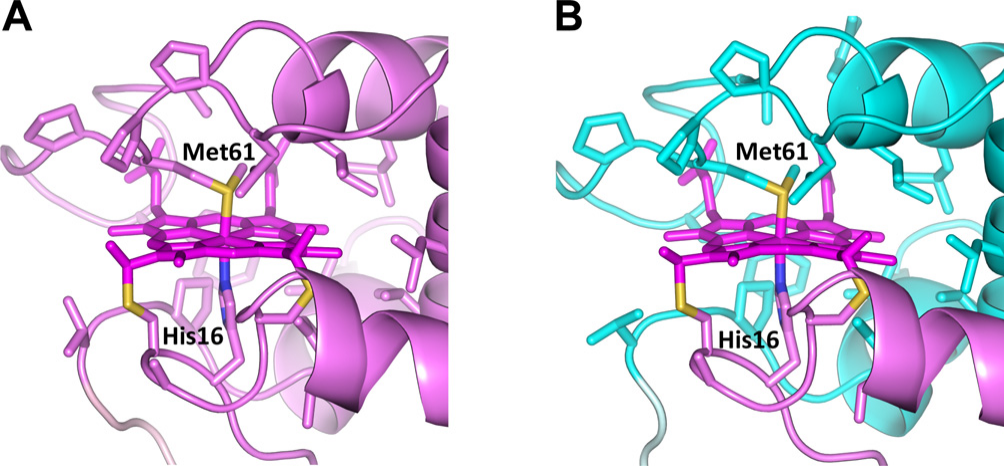
Active site structures of monomeric and dimeric WT PA cyt *c*_551_. (A) Structure of monomeric WT PA cyt *c*_551_ (PDB ID: 351C). (B) Structure of dimeric WT PA cyt *c*_551_ (PDB ID: 3X39). The heme and side-chains of amino acid residues near the heme (Phe7, Cys12, Ala14, Cys15, His16, Val23, Pro25, Val30, Leu44, Arg47, Ile48, Ser52, Trp56, Pro60, Met61, Pro62, Pro63, Asn64, Leu74, and Val78) are shown as stick models. The sulfur atoms of the heme axial Met ligand and heme-linked Cys are shown in yellow, and the nitrogen atoms of the heme axial His ligand are shown in blue. The cyan strand in the dimeric structure is a region from another molecule. The hemes and Thr20–Met22 residues (hinge loop) are depicted in dark and pale colors, respectively.

We solved the X-ray crystal structure of dimeric WT PA cyt *c*_551_ at 1.5 Å resolution to elucidate its detailed structure (Fig 2). There were two independent cyt *c*_551_ molecules with similar tertiary structures in an asymmetric unit of the crystal. Both cyt *c*_551_ molecules exhibited domain-swapped structures, where the N-terminal Glu1–Met22 (helix 1 and loop 1) and the heme were relocated from the original position observed in the monomer. The produced vacant area was occupied by Glu1–Asp19 (helix 1 and half of loop 1) from the other cyt *c*_551_ molecule. The hinge loop was constructed with only three amino acids; Thr20, Lys21, and Met22 (S4 Fig). Interestingly, the active site structure of the dimer was formed by the same amino acids as that of the monomer, but the heme axial ligands (His and Met) belonged to different protomers (Fig 3). The swapped region (Glu1–Asp19) and active site structure of dimeric PA cyt *c*_551_ were similar to those of dimeric HT cyt *c*_552_ [18], but different from those of dimeric horse cyt *c*, in which the Met80–heme iron bond was disrupted [15].

We have calculated the root-mean-square deviation (rmsd) for the Cα atoms of the regions from the N-terminus to Asp19 and from Val23 to the C-terminus between the structures of the monomer and each protomer (protomer 1 and protomer 2) of the dimer (S1 Table). The rmsd values of both regions were less than 0.9 Å (S5 Fig). These results indicate that the structures of both regions were similar between the monomer and each protomer of the dimer. The hydrogen bond network was also similar between the monomer and those of the dimer. The positions of the side-chains forming the proposed folding nucleus in cyt *c* family proteins (Pro3, Phe7, Leu74 and Trp77 in PA cyt *c*_551_) [47] did not change by the dimerization. There were four major hydrogen bonds (< 3.2 Å between heavy atoms; Cys15CO/Gly24NH, His16N_δ_/Pro25CO, Ala17CO/Tyr27NH, and Ile18CO/Lys28NH) between the N-terminal region (Glu1–Asp19) and the rest of the protein in the monomer (PDB ID: 351C) (S6 Fig). These hydrogen bonds were retained in the dimer, but were formed between different protomers (S6 Fig). In addition to these hydrogen bonds, a new hydrogen bond between loop 1 and helix 2 (Asp19_A_O_δ_/Lys28_B_N_ζ_) stabilized the dimer (S6 Fig). In the C-terminal region at loop 3, four intramolecular hydrogen bonds seen in the monomer (Ile48CO/Asn64N_δ_, Lys49CO/Asn64NH, Ser52CO/Met61NH, and Ser52NH/ Met61CO) were conserved in the dimer. The Fe–His16 and Fe–Met61 bond distances were also similar between the monomer and dimer (Table 2).

**Table 2:** Fe–His16 and Fe–Met61 distances in monomeric and dimeric WT PA cyt *c*_551_.

	Fe–His16 (Å)	Fe–Met61 (Å)
Monomer^a^	1.99	2.36
Dimer^b^	2.03	2.32
	2.07	2.30

^a^ PDB ID: 351C.

^b^ There are two independent WT PA cyt *c*_551_ molecules in the asymmetric unit of dimeric WT PA cyt *c*_551_ crystal.

### Effects of Met61 replacement with Ala on dimer formation of cytochrome *c*_551_

**Fig 4:**
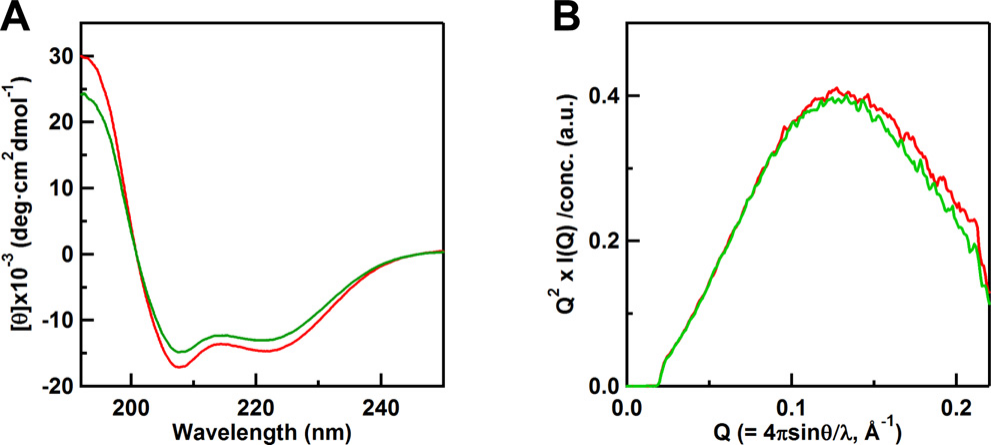
CD spectra and small angle X-ray scattering curves of WT and M61A PA cyt *c*_551_. (A) CD spectra of oxidized monomeric WT (red) and M61A (green) PA cyt *c*_551_. Measurement conditions: Sample concentration, 10 μM (heme unit); buffer, 50 mM potassium phosphate buffer; pH, 7.0; temperature, room temperature. (B) Small angle X-ray scattering curves of oxidized monomeric WT (red) and M61A (green) PA cyt *c*_551_ shown by Kratky plots. The intensities are normalized at their maximum intensities. Measurement conditions: sample concentration, 500 μM (heme unit); buffer, 50 mM potassium phosphate buffer; pH, 7.0; temperature, 20°C.

We replaced the heme-ligating Met61 of PA cyt *c*_551_ with Ala (M61A PA cyt *c*_551_) to investigate the effect of Met61 on oligomerization. The Soret band of oxidized monomeric WT PA cyt *c*_551_ at 409 nm blue shifted to 401 nm in the oxidized monomeric M61A PA cyt *c*_551_ spectrum (S7 Fig). The intensities of the negative 208-nm and 222-nm CD bands of oxidized M61A PA cyt *c*_551_ decreased by about 10% from those of the corresponding bands of oxidized WT PA cyt *c*_551_ (Fig 4A), indicating that the α-helical content of M61A PA cyt *c*_551_ decreased slightly compared to that of the WT protein. The radii of gyration were obtained as 13.7 and 13.9 Å for WT and M61A PA cyt *c*_551_, respectively, by SAXS measurements (Fig 4B). Although the size of the global structure of PA cyt *c*_551_ did not change significantly by the removal of Met61, the secondary structures were slightly perturbed (Fig 4). It has been reported that carboxylmethylation of Met61 of PA cyt *c*_551_ destabilizes its folded state [48], indicating that the α-helical structure of PA cyt *c*_551_ is stabilized by the Met–heme coordination. The amount of dimer produced by the treatment with ethanol decreased to less than 5% and no trimer or tetramer was detected for M61A PA cyt *c*_551_ (S8 Fig). These results indicate that the removal of heme-ligating Met in PA cyt *c*_551_ suppressed formation of oligomers by domain swapping.

### Differential scanning calorimetry measurement of dimeric cytochrome *c*_551_

DSC thermograms of oxidized monomeric and dimeric WT PA cyt *c*_551_ are shown in S9 Fig. The peak at 82°C for both the monomer and dimer corresponds to unfolding of the protein. No signal was observed below 82°C in the thermogram of the dimer, as well as that of the monomer. According to the results of gel chromatography (S1 Fig), the dimer dissociated to monomers when heated at 70°C for 10 min. These results show that the PA cyt *c*_551_ dimer dissociates to monomers without a significant enthalpy change (Δ*H* = ~0 kcal/mol). In the case of horse cyt *c*, the Δ*H* value for dimer-to-monomer transition was -40 ± 2 kcal/mol. Since Met did not dissociate from the heme iron in dimeric PA cyt *c*_551_ but dissociated in dimeric horse cyt *c*, we may attribute the difference between PA cyt *c*_551_ and horse cyt *c* in the enthalpy change on dimer dissociation to the difference in the heme coordination structure of the dimers.

### Redox potential of dimeric cytochrome *c*_551_

We measured the redox potential of dimeric PA cyt *c*_551_ by cyclic voltammetry to investigate the effect of domain swapping on the function. The midpoint redox potentials of monomeric and dimeric WT PA cyt *c*_551_ were obtained as 274 ± 5 and 242 ± 5 mV (vs NHE), respectively, at pH 7.0 in the presence of 200 mM NaCl (S10 Fig). The value for the monomer was similar to the reported value (276 ± 2 mV (vs NHE) in pH 7.0 at 25°C) [49]. Although the redox potential of the dimer decreased by about 30 mV compared to that of the monomer, the dimer exhibited a relatively high redox potential, which is characteristic for cyt *c* family proteins [50,51,52].

## Discussion

Met–heme coordination contributes to the stability of the structure and the ability of electron transfer in cyt *c* family proteins [48,53,54,55]. Although the optical absorption spectra and redox potentials were similar between monomeric and dimeric WT PA cyt *c*_551_, heme-ligating His and Met originated from different protomers in the dimer (Fig 3), similar to the case of dimeric HT cyt *c*_552_ [18]. In the case of dimeric horse cyt *c*, Met-heme coordination was perturbed and a hydroxide ion or a water molecule was coordinated to the heme iron [15]. The difference in the heme coordination structure between dimeric PA cyt *c*_551_ and dimeric horse cyt *c* may be due to the differences in the stability of the Met–heme coordination bond [26,27,28] and the rigidity of the loop containing the heme-ligating Met [20,29]. According to DSC measurements, Δ*H* for the dissociation of dimeric horse cyt *c* to monomers exhibited a large, negative value (-40 kcal/mol) [15], whereas the Δ*H* values for the dissociation of dimeric PA cyt *c*_551_ and dimeric HT cyt *c*_552_ were ~0 and +14 kcal/mol, respectively (S9 Fig) [18]. These results show that the coordination of Met to the heme contributes to stabilization of the dimer enthalpically.

Since carboxymethylation of Met61 destabilizes considerably the native state of PA cyt *c*_551_ [48], one may expect that removal of Met61 destabilizes dimeric PA cyt *c*_551_ and thus leads to an increase in high order oligomers. However, formation of dimers was minimal and no trimer was detected by the treatment of M61A PA cyt *c*_551_ with ethanol (S8 Fig). The α-helical structure of M61A PA cyt *c*_551_ was partially perturbed by the disruption of the Met–heme coordination bond (Fig 4A), although its global structure did not unfold completely (Fig 4B). Therefore, the intermolecular interactions through the N- and C-terminal α-helices and formation of high order oligomers may be suppressed by the perturbation of the α-helical structures in M61A PA cyt *c*_551_. The interaction between the N- and C-terminal α-helices in dimeric PA cyt *c*_551_ was similar to that of the monomer (Fig 2), although the swapping regions of PA cyt *c*_551_ was different from that of horse cyt *c*. We have shown that domain-swapped oligomers are generated by intermolecular hydrophobic interaction between the N- and C-terminal α-helices at the early stage of folding for horse cyt *c* [36]. These results indicate that the swapping region is defined subsequent to formation of the intermolecular interaction between the terminal α-helices which occurs during folding in cyt *c* family proteins. Moreover, the contacts between the N- and C-terminal α-helices are essential for not only protein folding but also domain swapping.

It has been suggested that PA cyt *c*_551_ folds through multiple transition states separated by a high energy intermediate [23], whereas HT cyt *c*_552_ transiently populates a compact obligatory intermediate during folding [56]. It has also been proposed that the propensity to form elements of stable secondary structures controls the process of folding in proteins [57,58]. It has been shown that the region with the highest helical propensity is helix 3 for PA cyt *c*_551_ according to the calculation by the program AGADIR [59], whereas it is helix 4 (C-terminal helix) for HT cyt *c*_552_ [25]. Although the highest helical propensity region differed between PA cyt *c*_551_ and HT cyt *c*_552_, the swapping regions in their dimers were similar, suggesting that the helical propensity does not define the swapping region.

**Fig 5:**
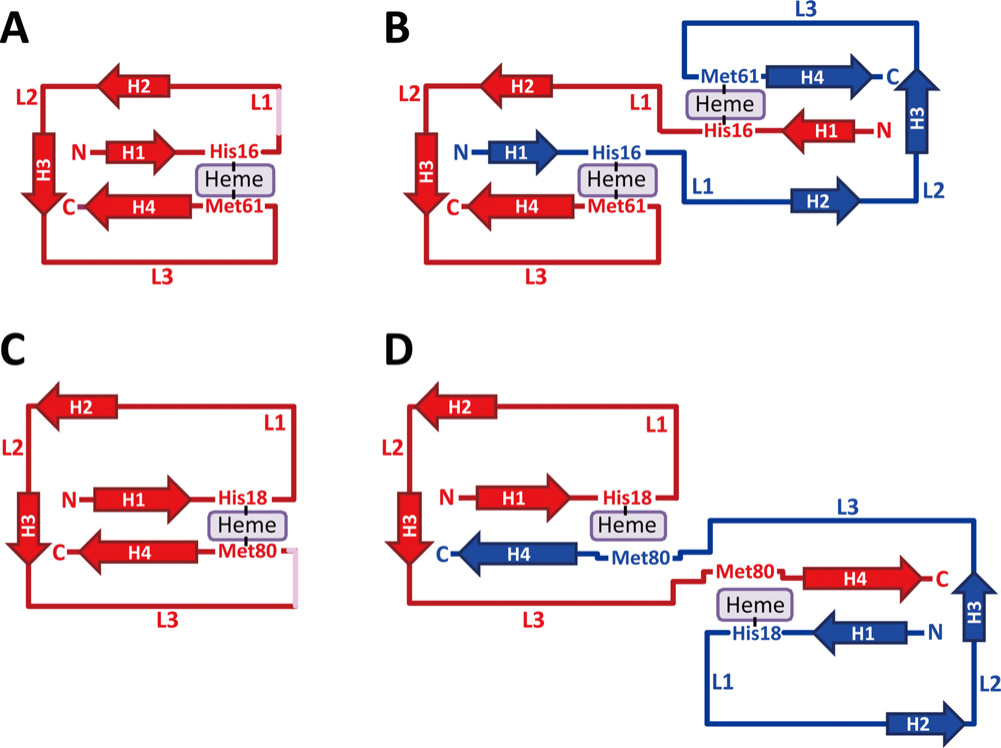
Topology diagrams of PA cyt *c*_551_ and horse cyt *c*. (A) Monomeric PA cyt *c*_551_, (B) dimeric PA cyt *c*_551_, (C) monomeric horse cyt *c*, and (D) dimeric horse cyt *c*. The helices and loops are labeled as H1–H4 and L1–L3, respectively. The helices are depicted as arrows. The hinge loops in the monomers are depicted in pink.

It has been suggested by molecular dynamics simulations that the native topology generally determines the domain-swapped structure [60]. Although the topology of the tertiary structure (Fig 5A and 5C) and folding features, such as the burst-phase collapse in the folding process, are similar between PA cyt *c*_551_ and horse cyt *c*, the swapping region was different between these proteins (Fig 5B and 5D); the hinge loops of PA cyt *c*_551_ and horse cyt *c* were Thr20–Met22 (at loop 1) and Thr78–Ala83 (at loop 3), respectively. According to hydrogen exchange NMR measurements, the thermodynamic property of foldons is different between PA cyt *c*_551_ and horse cyt *c* [20,21,24]. A foldon with low energy (small Δ*G*_HX_) loses its secondary structure at the early stage of unfolding, showing that the region corresponding to the low energy foldon has low structural stability. The region containing loop 1 and helix 2 of PA cyt *c*_551_ is low in stability [21], whereas that of loop 3 of horse cyt *c* is low [20,24]. Interestingly, the hinge loops of the domain-swapped structure in PA cyt *c*_551_ and horse cyt *c* correspond to the low stability regions of their monomers. Unfolding simulations have suggested that the hinge loop are ‘hot-spots’, around which proteins tend to locally unfold prior to complete unfolding [60]. These results show that the region with low stability in the monomer correlates to the hinge loop in domain swapping.

In summary, we found that the region containing the N-teminal α-helix and heme was exchanged in the domain-swapped dimer of PA cyt *c*_551_. The active site structure of the dimer was formed by the same amino acids as that of the monomer, but the heme axial ligands (His and Met) belonged to different protomers. By comparison of the domain-swapped structures of cyt *c* family proteins, we propose that the stability of the local structure may determine the position of the hinge loop in domain swapping, and thus the knowledge on protein folding may help to predict the structural features of domain swapping.

## Supporting Information

S1 Fig Elution curves of WT PA cyt *c*_551_. (A) Elution curve after an addition up to 80% (v/v) ethanol, subsequent lyophilization, and resolvation with buffer. (B) Elution curve of monomeric WT PA cyt *c*_551_. (C and D) Elution curves of the solution (C) before and (D) after heating purified dimeric WT PA cyt *c*_551_ at 70°C for 10 min. Absorbances at 409 nm (red) and 280 nm (blue) were detected. Measurement conditions: column, Superdex 75 10/300 GL column; flow rate, 0.5 ml/min; buffer, 50 mM potassium phosphate buffer; pH, 7.0; temperature, 4°C.
(TIF)

S2 Fig Optical absorption spectra of monomeric and dimeric WT PA cyt *c*_551_. Optical absorption spectra of oxidized monomeric (red) and dimeric (blue) WT PA cyt *c*_551_ are depicted for the (A) 250–800 nm and (B) 600–800 nm regions. Measurement conditions: sample concentration, (A) 7.6 μM and (B) 41 μM (heme unit); buffer, 50 mM potassium phosphate buffer; pH, 7.0; temperature, room temperature.
(TIF)

S3 Fig CD spectra of monomeric and dimeric WT PA cyt *c*_551_. CD spectra of oxidized monomeric (red) and dimeric (blue) WT PA cyt *c*_551_ are depicted. Concentration of each protein was calculated from the intensity of its Soret band. Measurement conditions were the same as those for S2 Fig, except for the sample concentration of 10 μM (heme unit).
(TIF)

S4 Fig Difference electron density map of dimeric WT PA cyt *c*_551_. The difference electron density map (*F*_obs_—*F*_calc_) superimposed on the Thr20–Met22 residues (hinge loop) of dimeric WT PA cyt *c*_551_ (pink and cyan) is depicted. The Thr20–Met22 residues were omitted from the calculations of the phases and structure factors (*F*_calc_). The electron density map is shown in green at a contour level of 2.5σ. The hemes and the Thr20–Met22 residues are depicted as stick models in dark and pale colors, respectively. The oxygen and nitrogen atoms of the Thr20–Met22 residues are shown in red and blue, respectively.
(TIF)

S5 Fig Superimposed structures of monomeric and dimeric WT PA cyt *c*_551_. Structures of monomeric (gray) and dimeric (pink and cyan) WT PA cyt *c*_551_ are superimposed. The hemes, Cys12, Cys15, His16, and Met61 are depicted as stick models. The Thr20–Met22 residues (hinge loop) are shown in pale colors. The hemes and Thr20–Met22 residues (hinge loop) are depicted in dark and pale colors, respectively. The sulfur atoms of the heme axial Met ligand and heme-linked Cys are shown in yellow, and the nitrogen atoms of the heme axial His ligand are shown in blue.
(TIF)

S6 Fig Major hydrogen bonds of WT PA cyt *c*_551_. Major hydrogen bonds (< 3.2 Ǻ between heavy atoms) between the N-terminal region and the rest of the protein in WT PA cyt *c*_551_ are depicted. (A) Hydrogen bonds of monomeric WT PA cyt *c*_551_: Cys15CO/Gly24NH, His16N_δ_/Pro25CO, Ala17CO/Tyr27NH, and Ile18CO/Lys28NH (PDB ID: 351C). (B) Hydrogen bonds of dimeric WT PA cyt *c*_551_: Cys15_A_CO/Gly24_B_NH, His16_A_N_δ_/Pro25_B_CO, Ala17_A_CO/Tyr27_B_NH, Ile18_A_CO/Lys28_B_NH, and Asp19_A_O_δ_/Lys28_B_N_ζ_ (PDB ID: 3X39). The N-terminal region (Gly1–Met22) and the rest of the protein are shown in pink and gray, respectively. The hemes, Cys12, Cys15, His16, Met61, and residues involved in the hydrogen bonds are shown as stick models. The hydrogen bonds are shown as dotted yellow lines. The nitrogen and oxygen atoms involved in the hydrogen bonds are shown in blue and red, respectively. The N- and C-termini are labeled as N and C, respectively.
(TIF)

S7 Fig Optical absorption spectra of WT and M61A PA cyt *c*_551_. Spectra of oxidized monomeric WT (red) and M61A (green) PA cyt *c*_551_ are depicted. Measurement conditions: sample concentration, 10 μM (heme unit); buffer, 50 mM potassium phosphate buffer; pH, 7.0; temperature, room temperature.
(TIF)

S8 Fig Elution curves of M61A PA cyt *c*_551_. (A) Elution curve after an addition up to 80% (v/v) ethanol, subsequent lyophilization, and resolvation with buffer. (B) Elution curve of monomeric M61A PA cyt *c*_551_. Absorbances at 409 (red) and 280 nm (blue) were detected. Measurement conditions were the same as those for S1 Fig.
(TIF)

S9 Fig Differential scanning calorimetry thermograms of monomeric and dimeric WT PA cyt *c*_551_. Thermograms of oxidized monomeric (red) and dimeric (blue) WT PA cyt *c*_551_ are depicted. Measurement conditions: sample concentration, 100 μM (heme unit); scan rate, 1°C/min; buffer, 50 mM potassium phosphate buffer; pH, 7.0.
(TIF)

S10 Fig Cyclic voltammograms of monomeric and dimeric WT PA cyt *c*_551_. Voltammograms of oxidized monomeric (red) and dimeric (blue) WT PA cyt *c*_551_ are depicted. Measurement conditions: sample concentration, 100 μM (heme unit); solvent, 50 mM potassium phosphate buffer containing 200 mM sodium chloride; pH, 7.0; temperature, room temperature; scan rate, 10 mV/s.
(TIF)

S1 Table Nucleotide sequences of the primers. (DOC)

S2 Table Statistics of data collection and structure refinement. (DOC)

S3 Table Root-mean-square deviation values between the structures of the monomer and protomers of the dimer. Root-mean-square deviation values for the Cα atoms of the N-terminal region and the rest of the protein (excluding the hinge loop) between the structures of the monomer and protomers of the dimer are calculated.
(DOC)
