# Supplementary figures and images for: Domain-Swapped Dimer of Pseudomonas aeruginosa Cytochrome c 551: Structural Insights into Domain Swapping of Cytochrome c Family Proteins
Source: PLoS One. 2015 Apr 8;10(4):e0123653. doi: 10.1371/journal.pone.0123653 (PMC4390240; doi:10.1371/journal.pone.0123653)

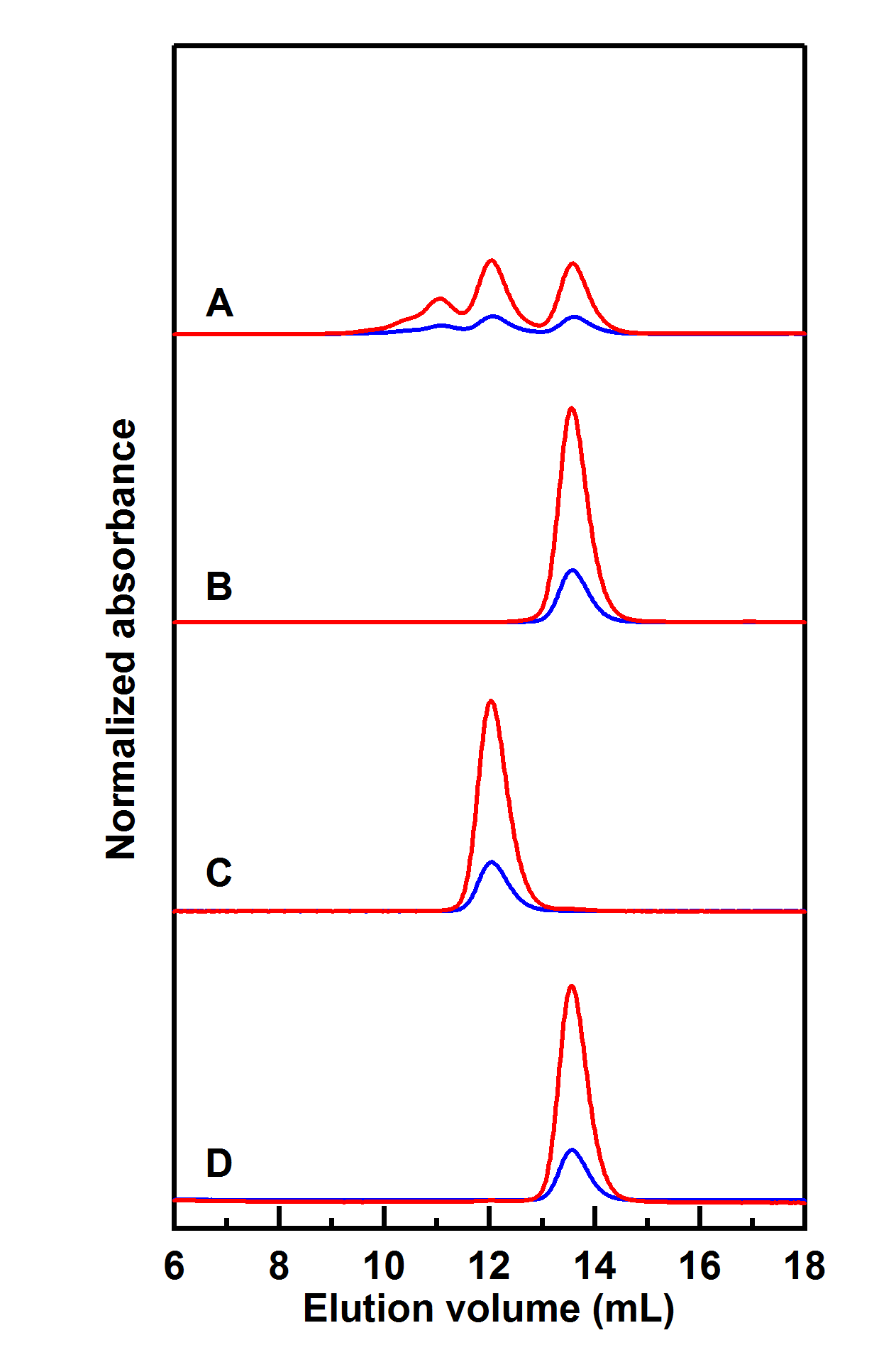

Supplement: S1 Fig — (A) Elution curve after an addition up to 80% (v/v) ethanol, subsequent lyophilization, and resolvation with buffer. (B) Elution curve of monomeric WT PA cyt c 551. (C and D) Elution curves of the solution (C) before and (D) after heating purified dimeric WT PA cyt c 551 at 70°C for 10 min. Absorbances at 409 nm (red) and 280 nm (blue) were detected. Measurement conditions: column, Superdex 75 10/300 GL column; flow rate, 0.5 ml/min; buffer, 50 mM potassium phosphate buffer; pH, 7.0; temperature, 4°C. (TIF) [file pone.0123653.s001.tif]

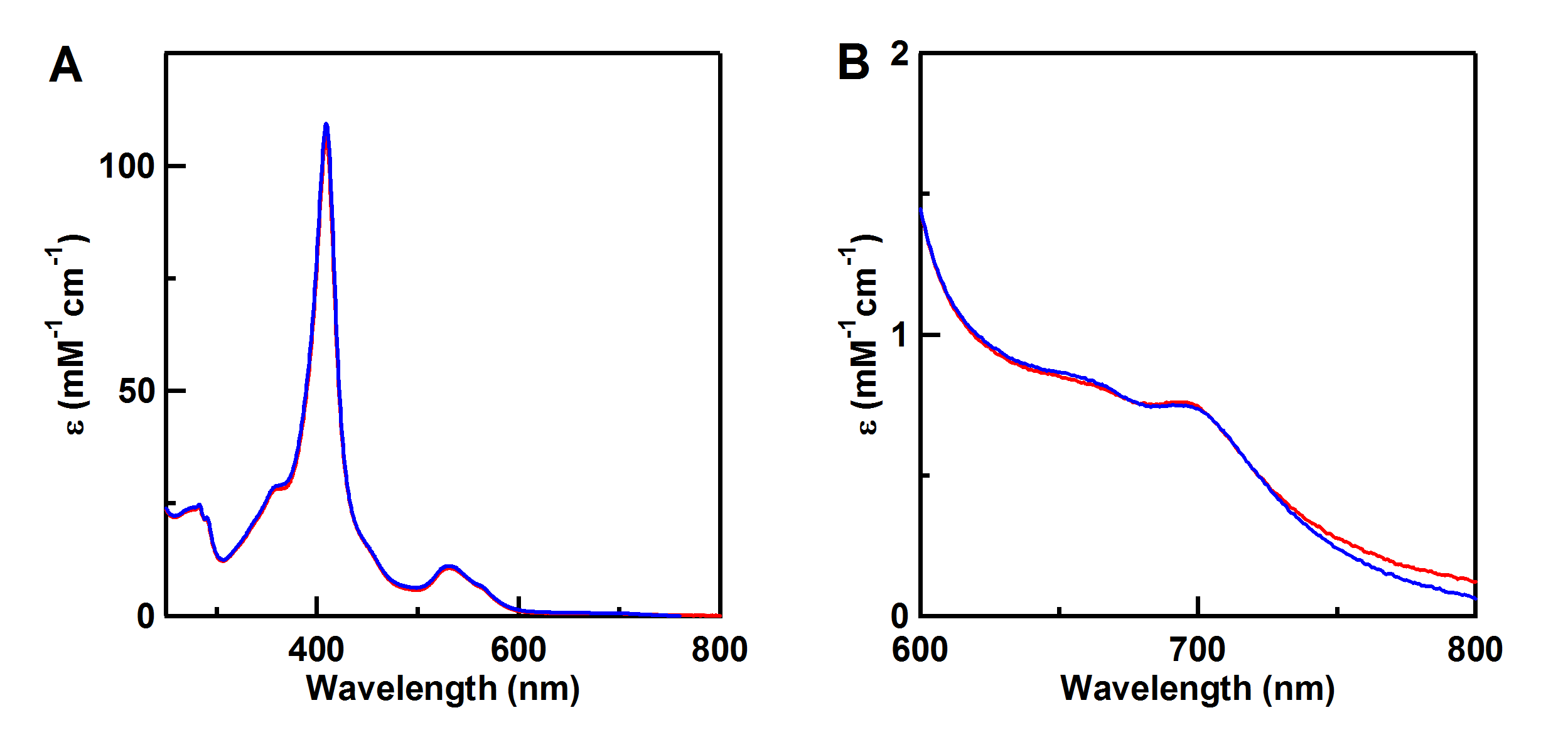

Supplement: S2 Fig — Optical absorption spectra of oxidized monomeric (red) and dimeric (blue) WT PA cyt c 551 are depicted for the (A) 250–800 nm and (B) 600–800 nm regions. Measurement conditions: sample concentration, (A) 7.6 μM and (B) 41 μM (heme unit); buffer, 50 mM potassium phosphate buffer; pH, 7.0; temperature, room temperature. (TIF) [file pone.0123653.s002.tif]

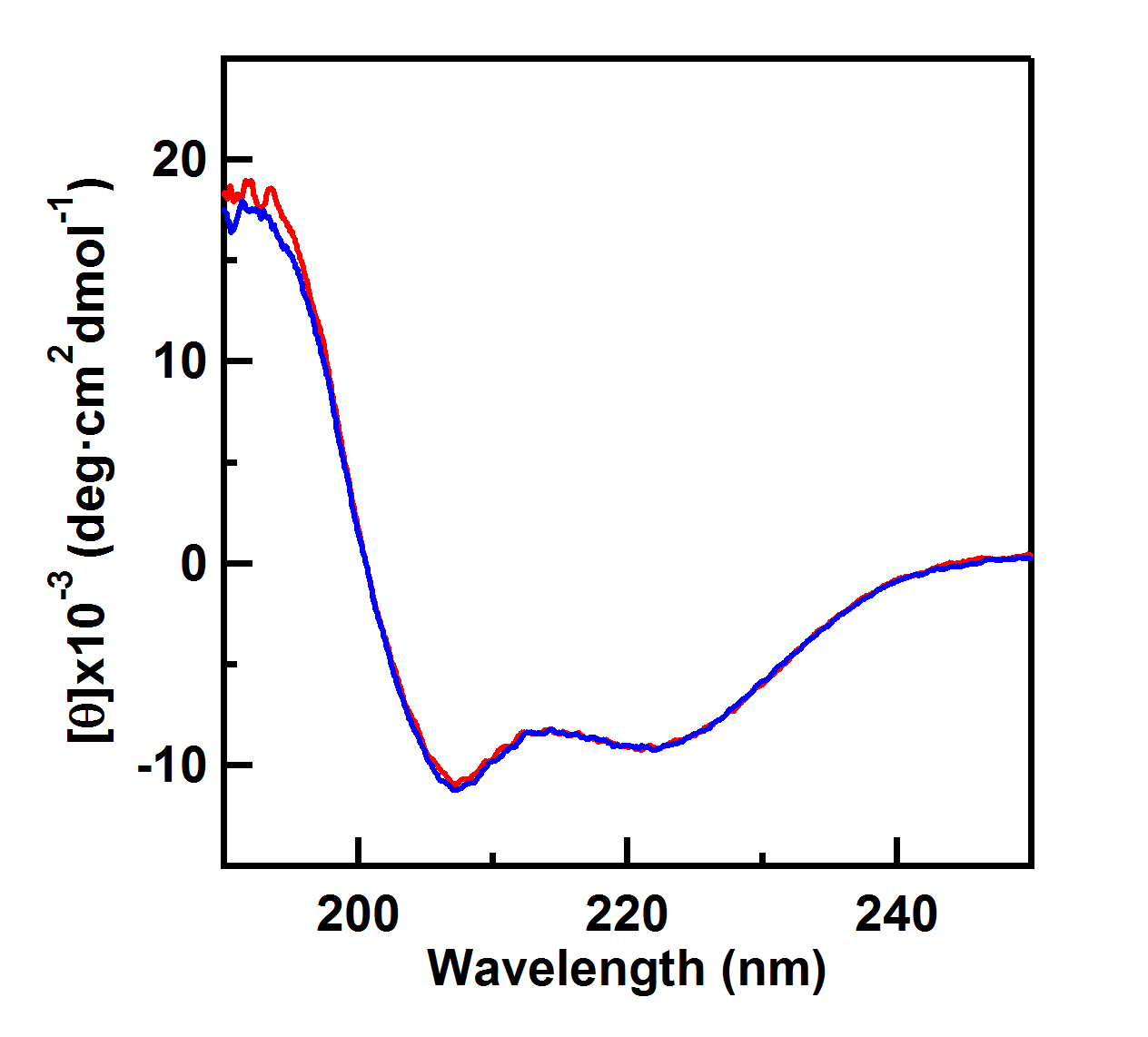

Supplement: S3 Fig — CD spectra of oxidized monomeric (red) and dimeric (blue) WT PA cyt c 551 are depicted. Concentration of each protein was calculated from the intensity of its Soret band. Measurement conditions were the same as those for S2 Fig, except for the sample concentration of 10 μM (heme unit). (TIF) [file pone.0123653.s003.tif]

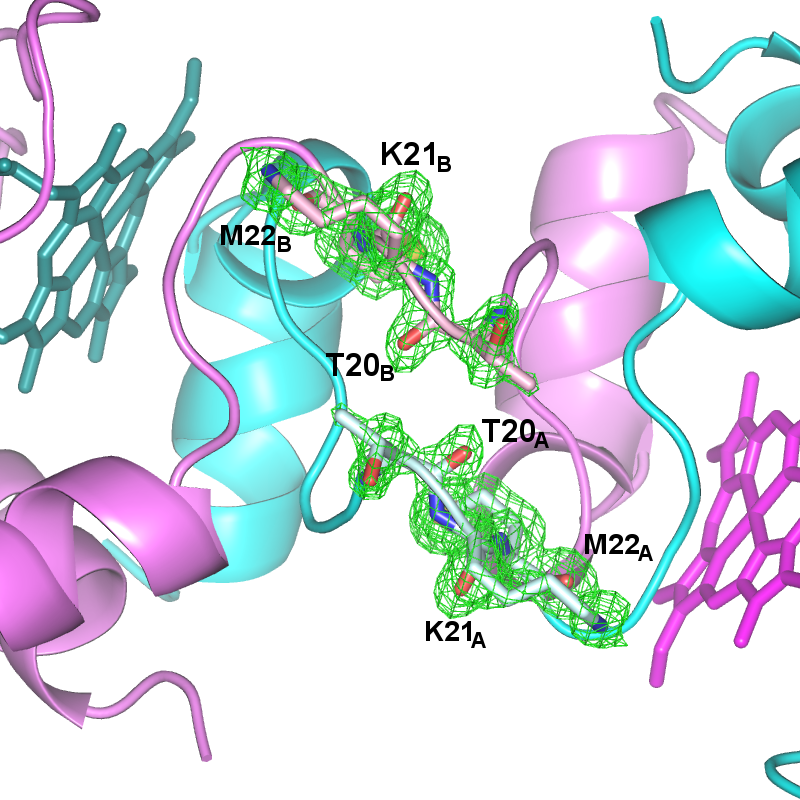

Supplement: S4 Fig — The difference electron density map (F obs—F calc) superimposed on the Thr20–Met22 residues (hinge loop) of dimeric WT PA cyt c 551 (pink and cyan) is depicted. The Thr20–Met22 residues were omitted from the calculations of the phases and structure factors (F calc). The electron density map is shown in green at a contour level of 2.5σ. The hemes and the Thr20–Met22 residues are depicted as stick models in dark and pale colors, respectively. The oxygen and nitrogen atoms of the Thr20–Met22 residues are shown in red and blue, respectively. (TIF) [file pone.0123653.s004.tif]

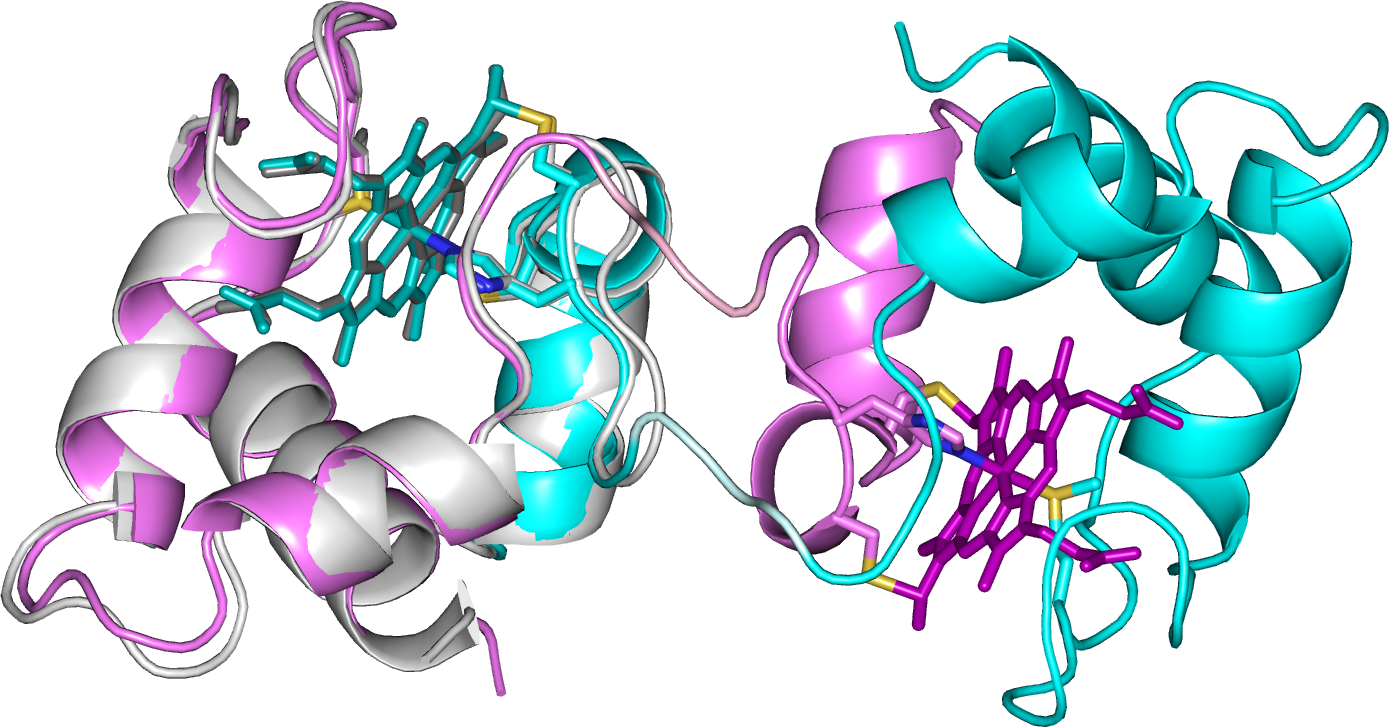

Supplement: S5 Fig — Structures of monomeric (gray) and dimeric (pink and cyan) WT PA cyt c 551 are superimposed. The hemes, Cys12, Cys15, His16, and Met61 are depicted as stick models. The Thr20–Met22 residues (hinge loop) are shown in pale colors. The hemes and Thr20–Met22 residues (hinge loop) are depicted in dark and pale colors, respectively. The sulfur atoms of the heme axial Met ligand and heme-linked Cys are shown in yellow, and the nitrogen atoms of the heme axial His ligand are shown in blue. (TIF) [file pone.0123653.s005.tif]

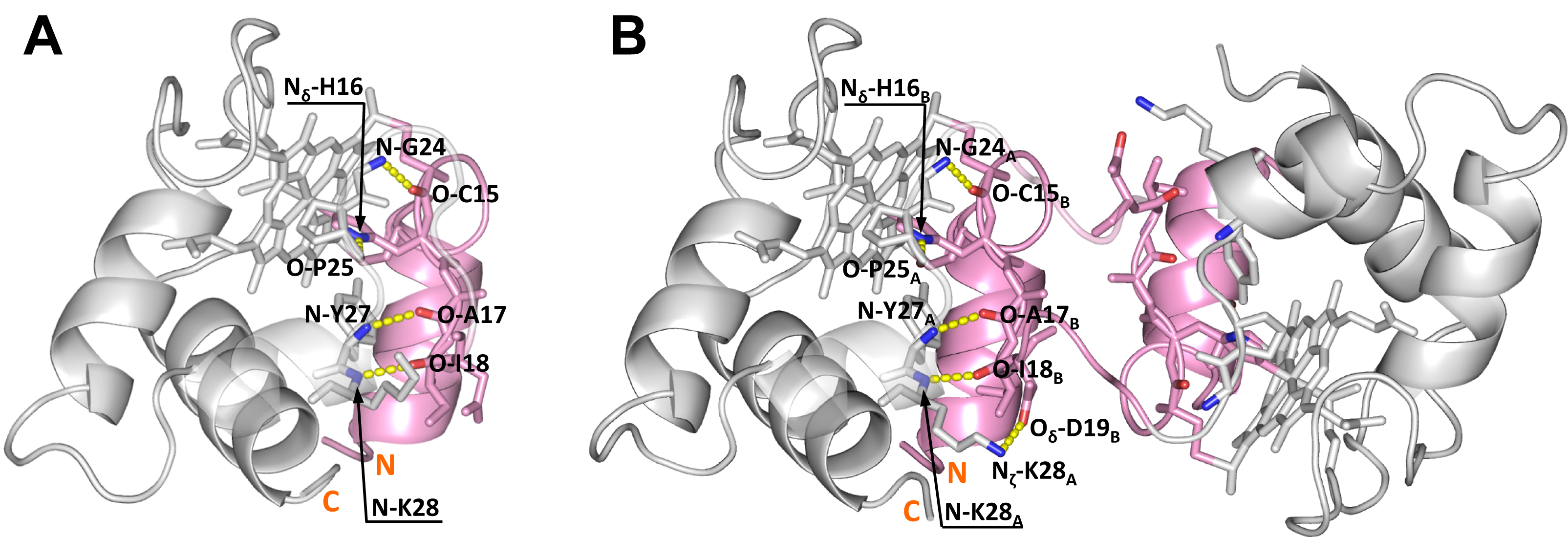

Supplement: S6 Fig — Major hydrogen bonds (< 3.2 Ǻ between heavy atoms) between the N-terminal region and the rest of the protein in WT PA cyt c 551 are depicted. (A) Hydrogen bonds of monomeric WT PA cyt c 551: Cys15CO/Gly24NH, His16Nδ/Pro25CO, Ala17CO/Tyr27NH, and Ile18CO/Lys28NH (PDB ID: 351C). (B) Hydrogen bonds of dimeric WT PA cyt c 551: Cys15ACO/Gly24BNH, His16ANδ/Pro25BCO, Ala17ACO/Tyr27BNH, Ile18ACO/Lys28BNH, and Asp19AOδ/Lys28BNζ (PDB ID: 3X39). The N-terminal region (Gly1–Met22) and the rest of the protein are shown in pink and gray, respectively. The hemes, Cys12, Cys15, His16, Met61, and residues involved in the hydrogen bonds are shown as stick models. The hydrogen bonds are shown as dotted yellow lines. The nitrogen and oxygen atoms involved in the hydrogen bonds are shown in blue and red, respectively. The N- and C-termini are labeled as N and C, respectively. (TIF) [file pone.0123653.s006.tif]

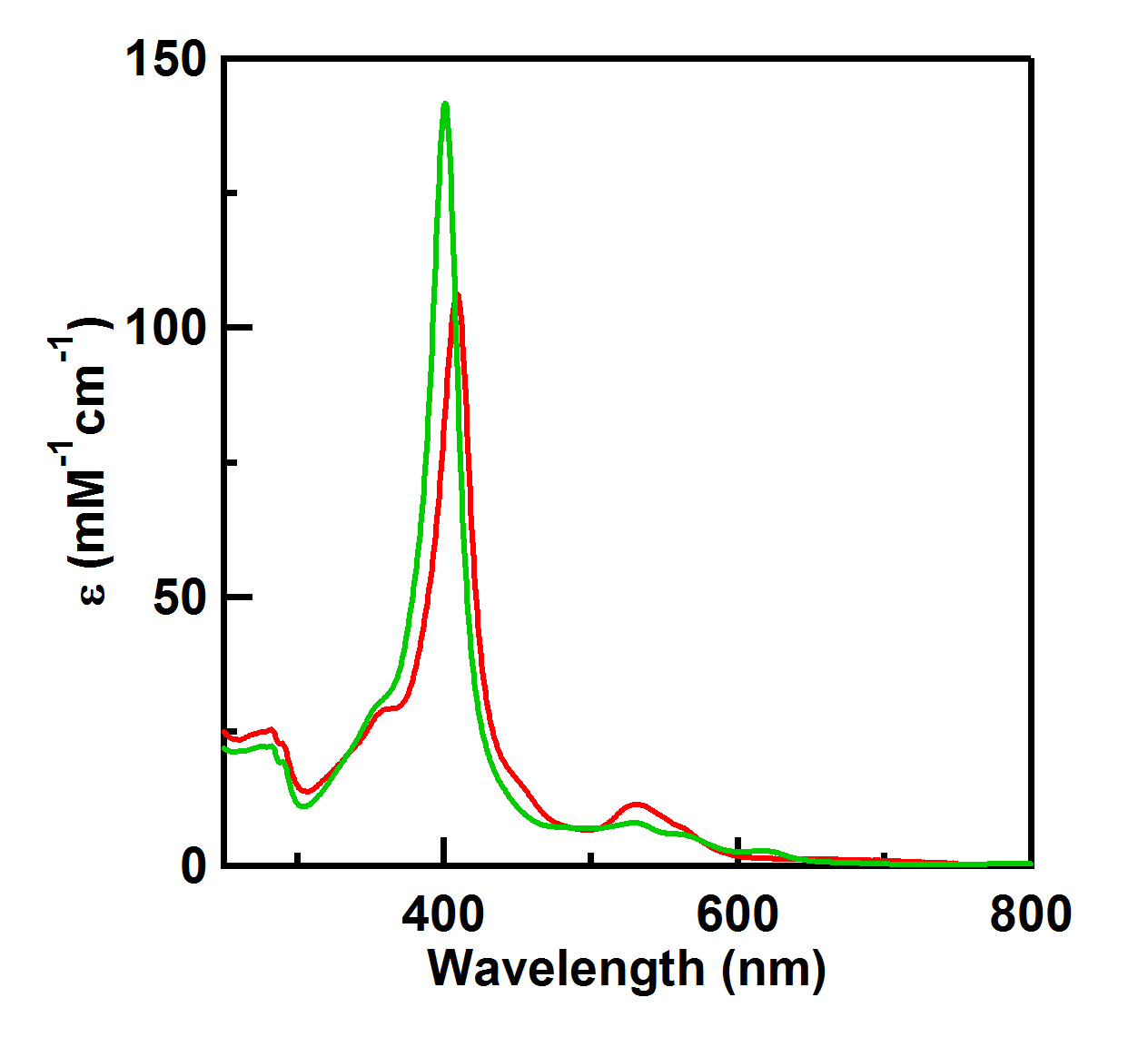

Supplement: S7 Fig — Spectra of oxidized monomeric WT (red) and M61A (green) PA cyt c 551 are depicted. Measurement conditions: sample concentration, 10 μM (heme unit); buffer, 50 mM potassium phosphate buffer; pH, 7.0; temperature, room temperature. (TIF) [file pone.0123653.s007.tif]

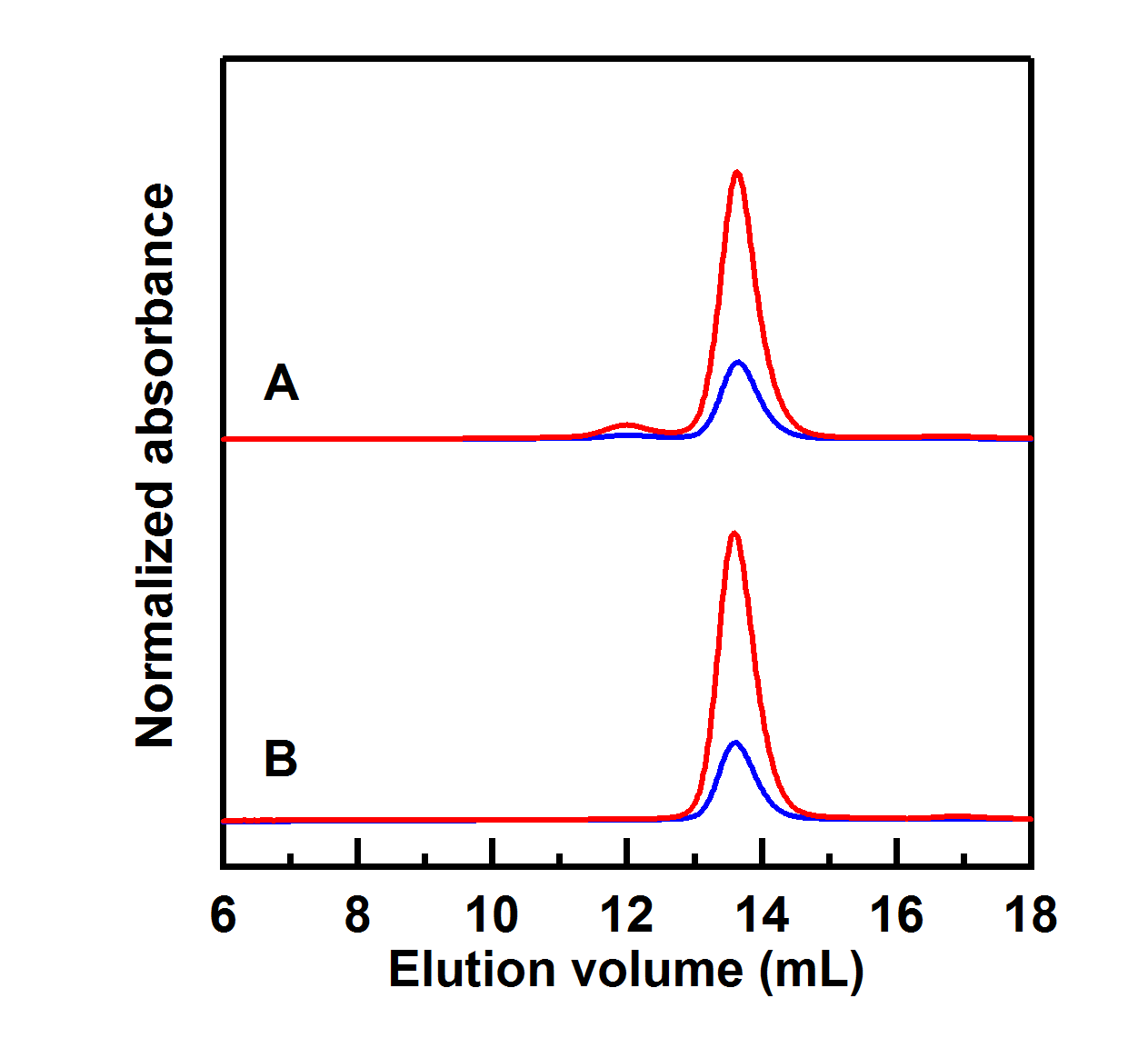

Supplement: S8 Fig — (A) Elution curve after an addition up to 80% (v/v) ethanol, subsequent lyophilization, and resolvation with buffer. (B) Elution curve of monomeric M61A PA cyt c 551. Absorbances at 409 (red) and 280 nm (blue) were detected. Measurement conditions were the same as those for S1 Fig. (TIF) [file pone.0123653.s008.tif]

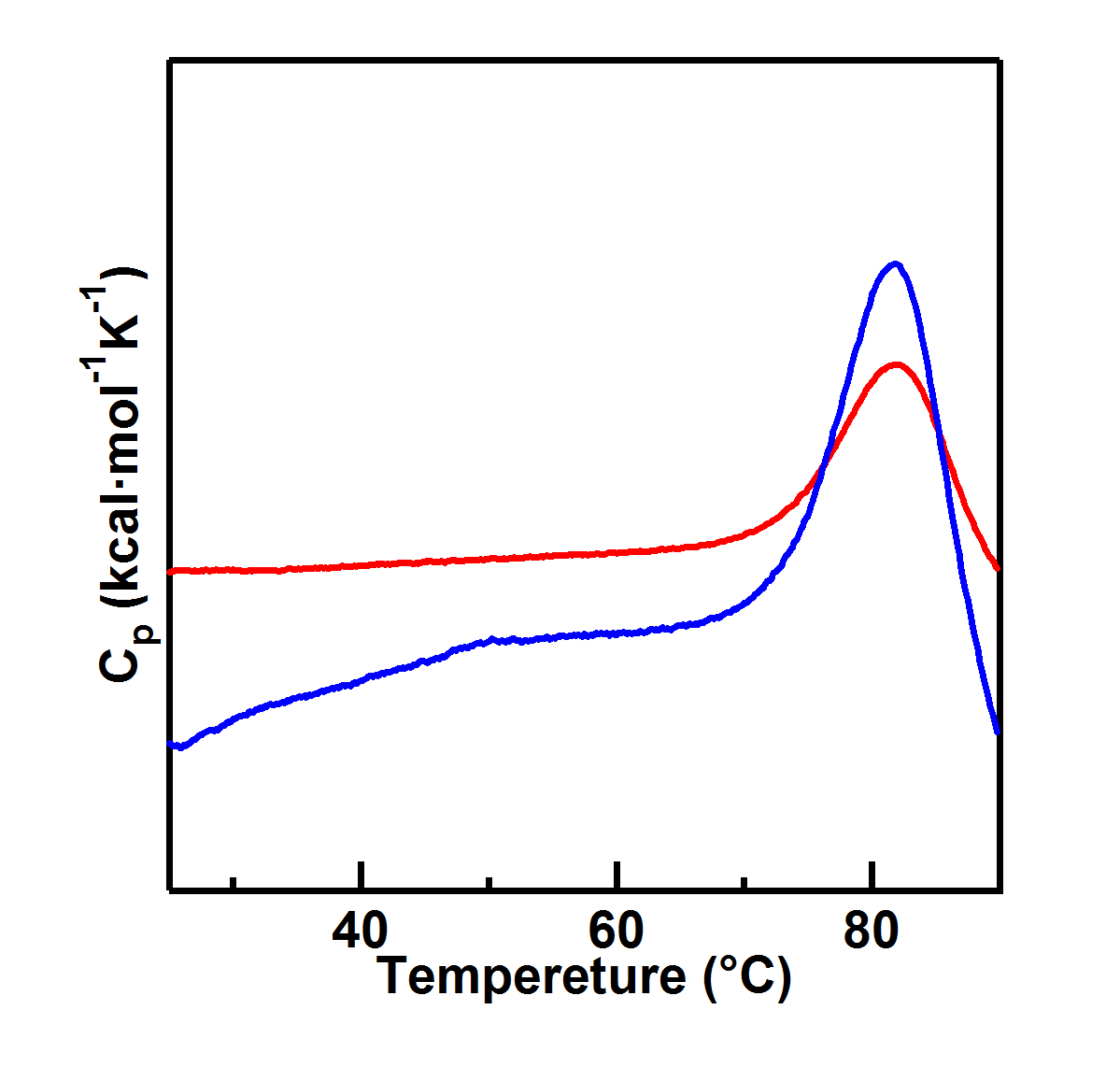

Supplement: S9 Fig — Thermograms of oxidized monomeric (red) and dimeric (blue) WT PA cyt c 551 are depicted. Measurement conditions: sample concentration, 100 μM (heme unit); scan rate, 1°C/min; buffer, 50 mM potassium phosphate buffer; pH, 7.0. (TIF) [file pone.0123653.s009.tif]

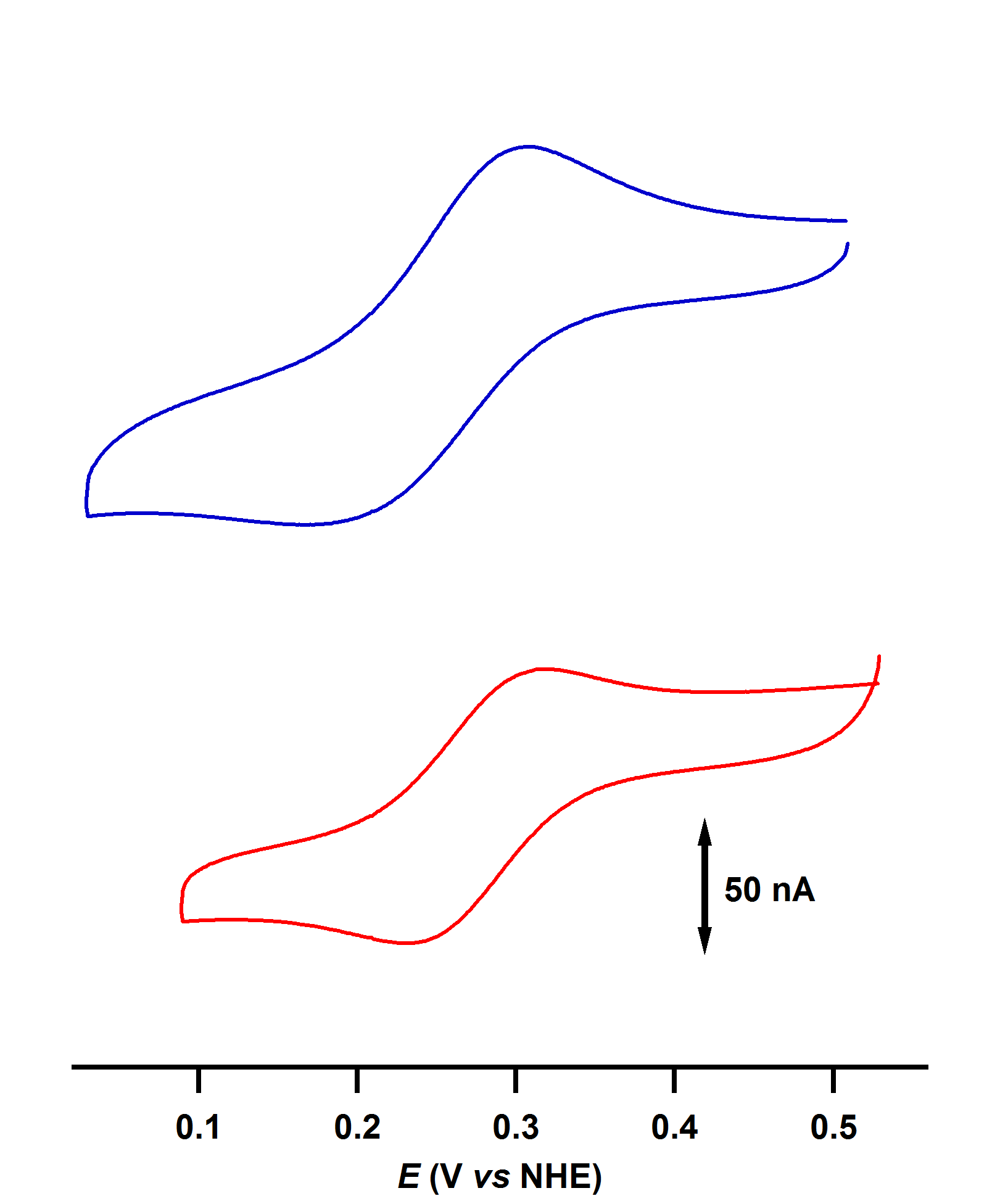

Supplement: S10 Fig — Voltammograms of oxidized monomeric (red) and dimeric (blue) WT PA cyt c 551 are depicted. Measurement conditions: sample concentration, 100 μM (heme unit); solvent, 50 mM potassium phosphate buffer containing 200 mM sodium chloride; pH, 7.0; temperature, room temperature; scan rate, 10 mV/s. (TIF) [file pone.0123653.s010.tif]
