# Supplementary material for: Domain-Swapped Dimer of Pseudomonas aeruginosa Cytochrome c 551: Structural Insights into Domain Swapping of Cytochrome c Family Proteins
Source: PLoS One. 2015 Apr 8;10(4):e0123653. doi: 10.1371/journal.pone.0123653 (PMC4390240; doi:10.1371/journal.pone.0123653)
Supplement: S1 Table — (DOC) [file pone.0123653.s011.doc]

| Primer Sequence^a^ |
| --- |
| PA-M61A-F GGCCCGATCCCGGCGCCGCCGAACGCG |
| PA-M61A-R CGCGTTCGGCGGCGCCGGGATCGGGCC |

^a^ Underlines indicate the nucleotides for the modified amino acid.
