# Supplementary material for: Domain-Swapped Dimer of Pseudomonas aeruginosa Cytochrome c 551: Structural Insights into Domain Swapping of Cytochrome c Family Proteins
Source: PLoS One. 2015 Apr 8;10(4):e0123653. doi: 10.1371/journal.pone.0123653 (PMC4390240; doi:10.1371/journal.pone.0123653)
Supplement: S2 Table — (DOC) [file pone.0123653.s012.doc]

| Data collection |  |
| --- | --- |
| X-ray source | SPring-8 (BL38B1) |
| Wavelength (Å) | 0.8000 |
| Space group  Unit cell parameters  *a*, *b*, *c* (Å)  α, β, γ (˚) | *P*6_3_22  82.6, 82.3, 89.8  90.0, 90.0, 120.0 |
| Resolution (Å)  Number of unique reflections  *R*_merge_^a^  Completeness (%)  <*I*/σ(*I*)>  Redundancy | 50.0-1.50 (1.55-1.50)  29583 (2893)  0.062 (0.551)  99.9 (100.0)  57.9 (7.03)  20.7 (20.6) |
|  |  |
| Refinement |  |
| Resolution (Å)  Number of reflections  *R*_work_^b^ (%)  *R*_free_^b^ (%)  Completeness (%) | 50.0-1.50 (1.54-1.50)  27950 (2007)  13.9 (12.9)  18.8 (21.9)  99.5 (99.6) |
| Number of atoms in an asymmetric unit  Protein  Water  Heme | 1236  191  86 |
| Average *B* factors (Å^2^)  Protein  Water  Heme | 28.1  35.8  17.7 |
| Ramachandran plot (%)  Favored region  Allowed region  Outlier region | 100.0  0.0  0.0 |

Statistics for the highest-resolution shell are given in parentheses.

^a^ *R*_merge_=Σ_hkl_ | *I* – <*I*> | (Σ_hkl_| *I* |)^−1^.

^b^ *R*_work_=Σ_hkl_ | | *F*_obs_ | – *k*| *F*_calc_ | | ( Σ_hkl_ | *F*_obs_ | ) ^−1^, *k*: scaling factor. *R*_free_ was computed identically, except where all reflections belong to a test set of 5 % of randomly selected data.
