# Supplementary material for: Domain-Swapped Dimer of Pseudomonas aeruginosa Cytochrome c 551: Structural Insights into Domain Swapping of Cytochrome c Family Proteins
Source: PLoS One. 2015 Apr 8;10(4):e0123653. doi: 10.1371/journal.pone.0123653 (PMC4390240; doi:10.1371/journal.pone.0123653)
Supplement: S3 Table — Root-mean-square deviation values for the Cα atoms of the N-terminal region and the rest of the protein (excluding the hinge loop) between the structures of the monomer and protomers of the dimer are calculated. (DOC) [file pone.0123653.s013.doc]

| N-terminal region^a^ (Å) | The rest of the protein^b^ (Å) |
| --- | --- |
| protomer 1 0.36  protomer 2 0.46 | 0.82  0.55 |

^a^1-19 amino acid residues.

^b^23-82 amino acid residues.
